# Immune response to COVID‐19 vaccination in patients with Waldenström macroglobulinaemia who pause their BTKi therapy

**DOI:** 10.1002/jha2.724

**Published:** 2023-06-21

**Authors:** Katherine Rankin, Priyanka Hastak, Alexander Wong, Sarah C. Sasson, Brendan Beaton, Avani Yeola, Andrew Warden, Stuart Turville, Anthony D. Kelleher, Fabienne Brilot, Judith Trotman

**Affiliations:** ^1^ Haematology Department Concord Repatriation General Hospital Sydney New South Wales Australia; ^2^ The Kirby Institute The University of New South Wales Sydney New South Wales Australia; ^3^ Concord Clinical School Faculty of Medicine and Health The University of Sydney Sydney New South Wales Australia; ^4^ Department of Clinical Immunology and Immunopathology ICPMR Westmead Hospital Sydney New South Wales Australia; ^5^ Brain Autoimmunity Group Kids Neuroscience Centre Kids Research The Children's Hospital at Westmead Sydney New South Wales Australia; ^6^ WMozzies: Australian Patient Support Group for Waldenström's Macroglobulinemia Sydney New South Wales Australia; ^7^ Sydney Institute of Infectious Disease The University of Sydney Sydney New South Wales Australia; ^8^ The School of Medical Sciences Faculty of Medicine and Health The University of Sydney Sydney New South Wales Australia

**Keywords:** lymphoma, vaccines, Waldenström macroglobulinaemia

## Abstract

Patients with Waldenström macroglobulinaemia (WM) are at increased risk of severe COVID‐19 infection and have poor immune responses to COVID‐19 vaccination. This study assessed whether a closely monitored pause in Bruton's Tyrosine Kinase inhibitor (BTKi) therapy might result in an improved humoral response to a 3rd COVID‐19 vaccine dose. Improved response was observed in WM patients who paused their BTKi, compared to a group who did not pause their BTKi. However, the response was attenuated after BTKi recommencement. This data contributes to our understanding of vaccination strategies in this patient group and may help inform consensus approaches in the future.

## 1 INTRODUCTION

Patients with indolent lymphoma are at increased risk of severe COVID‐19 disease [1]. Waldenström macroglobulinaemia (WM) is a rare type of non‐Hodgkin's lymphoma characterised by malignant proliferation of lymphoplasmacytoid cells and monoclonal IgM secretion [2]. Patients with WM are at increased risk of SARS‐CoV‐2 infection due to underlying disease features and treatment related immunosuppression [3].

Bruton's Tyrosine Kinase inhibitors (BTKi) are utilised in the treatment of haematological malignancies including WM. These drugs irreversibly bind the BTK protein, impairing the B cell receptor signalling pathway and have a short therapeutic half‐life [4]. It has been previously demonstrated that patients on BTKi therapy have reduced immunological responses to vaccination [5, 6]. Several recent studies have shown poor response to COVID‐19 vaccination in patients treated with BTKi for chronic lymphocytic leukaemia [7, 8]. We previously reported poor generation of anti‐spike IgG antibodies and low levels of live virus neutralisation in a prospective study of two doses of an mRNA COVID‐19 vaccine in patients with WM on BTKi therapy [9]. However, COVID‐specific T cell responses were preserved [9]. These findings have been confirmed in other studies [10, 11].

Since our cohort was recruited, a 3rd COVID‐19 vaccine was recommended to complete primary vaccination and provided further opportunity to assess, and potentially improve, vaccine immunogenicity. We hypothesised that a short pause in BTKi therapy might eventuate in a period of time where the patient's response to COVID‐19 vaccination could be enhanced.

## 2 MATERIALS AND METHODS

The prospective TReatment Interruption of BTKi to Enhance COVID‐19 Antibody response (TRIBECA) study was designed to assess anti‐Spike IgG and virus neutralization responses to a 3rd COVID‐19 vaccine dose in patients with WM willing to undergo a monitored pause in their BTKi therapy (*N* = 9) prior to and after the 3rd dose. The patients were originally recruited to a larger parent study commencing in 2021, which examined initial immune response to the first two COVID‐19 vaccine doses. They were given information on the risks and benefits of pausing BTKi therapy and were subsequently given the option of participating in TRIBECA. We compared their response to WM patients in the parent study remaining on continuous BTKi (*N* = 8). Patients with prior COVID‐19 infection were not excluded. The study was approved by the Sydney Local Health District Human Research Ethics Committee and all subjects provided written informed consent prior to participation.

Immune response was measured prior to and up to 28 days after a 3rd vaccine dose, administered between October 2021 and February 2022. Immune response was also measured, in BTKi pause participants, up to 77 days after resuming BTKi. Humoral response to spike was measured by mean fluorescence intensity (MFI) of anti‐SARS‐CoV‐2 spike antibodies (ASAb), obtained using a high‐sensitivity flow cytometry cell assay, and live virus neutralization to a panel of SARS‐CoV‐2 variants of concern [12, 13].

Patients in the TRIBECA study paused their BTKi 3–4 days prior to the 3rd vaccine dose and recommenced therapy at a median of 24 days (range 18–31 days) afterwards, depending on their willingness and tolerance of treatment interruption. Patients were closely monitored during the BTKi pause with weekly clinical assessments for symptoms of disease progression as well as measurement of full blood count and IgM.

Statistical analysis of median MFI and virus neutralisation was performed using the non‐parametric Mann–Whitney test as well as one way ANOVA test. Prism software was used (Graphpad, San Diego, USA). A *p* value <0.05 was considered statistically significant.

## 3 RESULTS

The median age in the BTKi pause group was 73 years, and six of nine were male. In the non‐pause group, median age was 71 years, and seven of eight were male. In the BTKi pause group, six of nine were on zanubrutinib and three of nine on ibrutinib. In the non‐pause group, five of eight were on zanubrutinib, and three of eight were on ibrutinib. All received mRNA based COVID‐19 vaccines (either mRNA‐1273 or BNT162b2). One patient in the BTKi pause group died of urosepsis one month after recommencing BTKi.

Median haemoglobin in the BTKi pause group prior to cessation was 131 g/L (range 118–157), dropping to a median of 124 g/L (range 108–144) at the time of BTKi recommencement and returning to 127 g/L (range 116–150) one month post recommencement. Median IgM prior to BTKi pause was 4.3 g/L (range 2.8‐27.6) and rose to a median of 19.9 g/L (range 9.9‐40.3) at time of recommencement with a fall to 12.8 g/L (range 2.7–36.1) one month post recommencement. IgM levels continued to fall over subsequent months in all nine patients (data not shown).

**FIGURE 1:**
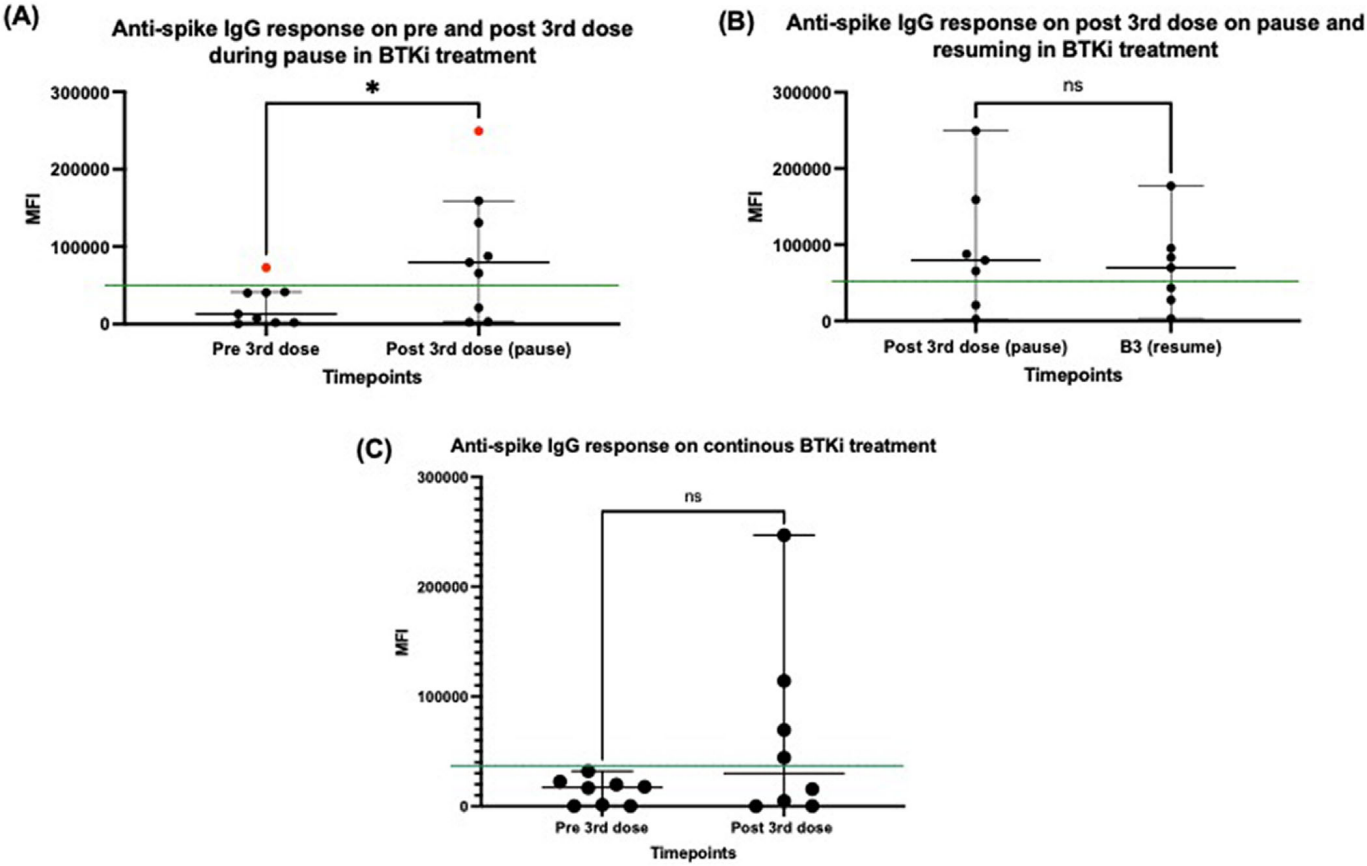
Comparison of anti‐spike IgG responses within groups using Mann–Whitney test. (A) Significant rise in median mean fluorescence intensity (MFI) in Bruton's Tyrosine Kinase inhibitor (BTKi) pause group following 3rd vaccine dose (*p* = 0.04). Red dots represent a patient who acquired COVID infection during the study period. Green line represents antibody positivity cutoff. (B) Non‐significant fall in median MFI in BTKi pause group following resumption of BTKi (*p* = 0.90) (C) Median MFI in non BTKi pause group did not significantly rise after the 3rd vaccine dose (*p* = 0.57).

ASAb median MFI pre 3rd dose in the BTKi pause group was 12878 (range 320—72,985) with a significant increase to 79,688 (range 2356—249,418) at time of BTKi recommencement 25 days (range 13–28) post 3rd dose (*p* = 0.04) (Figure 1A). In the WM patients on continuous BTKi, the median MFI pre 3rd dose of 17243 (range 206—31,972) was not significantly different to the median MFI 28 days (range 21–40) post 3rd dose of 30033 (range 125—246,899) (*p* = 0.57). (Figure 1C).

Seven patients in the BTKi pause group had a repeat ASAb response measured up to 77 days after BTKi recommencement and at this time the median MFI fell to 69810 (range 3109 −177,290) (Figure 1B). One patient in this group acquired a COVID‐19 infection between recommencing BTKi and having their post BTKi recommencement MFI measured. Another patient had clinical symptoms suspicious of COVID‐19 infection, although no positive COVID‐19 test result was recorded.

**FIGURE 2:**
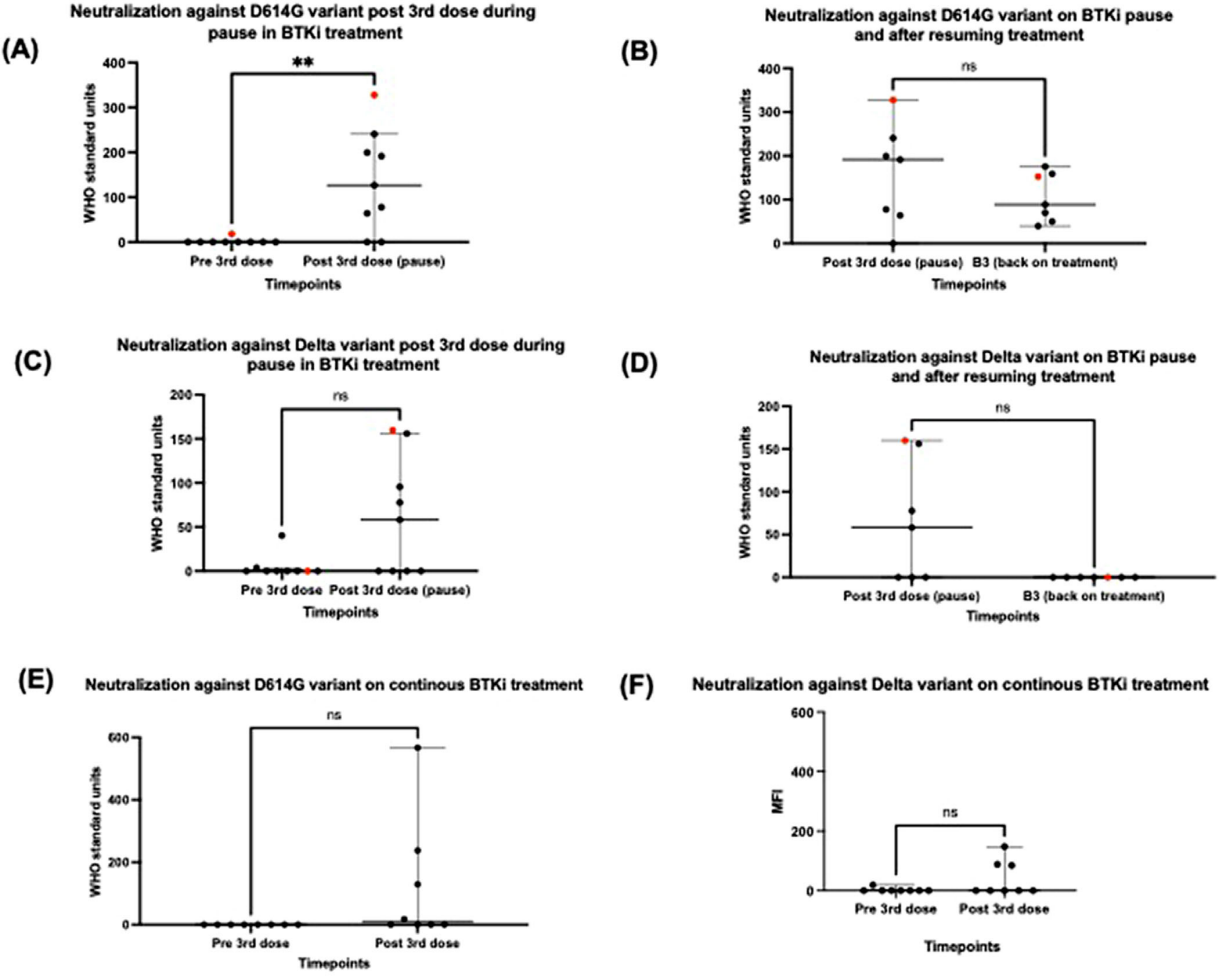
Comparison of virus neutralization (VN) results using Mann–Whitney test. (A) Significant increase in VN to D614G in Bruton's Tyrosine Kinase inhibitor (BTKi) pause group following 3rd vaccine dose (*p* < 0.05). (B) Non‐significant fall in VN to D614G after BTKi resumption (*p* = 0.32) (C) Non‐significant increase in VN to delta in BTKi pause group following 3rd vaccine dose (*p* = 0.06). (D) Non‐significant fall in VN to delta after BTKi resumption (*p* = 0.07). (E) Non‐significant rise in VN to D614G in continuous BTKi group (*p* = 0.08) (F) Non‐significant rise in VN to delta in continuous BTKi group (*p* = 0.2).

BTKi treatment interruption was associated with significant improvement in neutralization to early clade and improvement (albeit non‐significant) to delta SARS‐CoV‐2 variant. One month after BTKi recommencement, a non‐significant decline was observed in neutralization to early clade and delta variant (Figure 2A–D). One way ANOVA was also used to compare differences in virus neutralisation across all three time points, and this produced statistically significant results for both early clade (*p* = 0.0035) and delta variants (*p* = 0.0048).

Virus neutralization was not as pronounced in the non BTKi pause patients with only three patients having an improvement to neutralization to early clade and two patients having improvement in neutralization to delta at one month post vaccination (Figure 2E,F).

## 4 DISCUSSION

Our study demonstrates that patients with WM who paused their BTKi therapy prior to and following 3rd dose of a COVID‐19 vaccine for a limited period of time showed a transiently improved humoral immune response. Patients who did not pause their BTKi did not achieve any improvement in their immune response. The improved humoral response in BTKi pause patients was partially reduced following BTKi re‐introduction, although there was persistence of neutralization to the early clade variant. Our ongoing work will determine the T cell responses in these groups.

In regards to safety, while there were the expected haemoglobin declines and IgM rises during BTKi cessation, no patient experienced symptomatic progression of their WM whilst off their BTKi, and the BTKi was promptly recommenced with subsequent biochemical improvement.

We acknowledge the limitations of this study including the relatively small patient numbers, heterogeneity in the patient population recruited and minor variation in pause duration. Additionally, the occurrence of COVID‐19 infection in some participants and the impact on subsequent immune response measurement needs to be taken into account.

Despite these limitations, this study charts an improvement in humoral response to COVID vaccination after a pause in BTKi therapy. While it may be reasonable to administer booster vaccination during scheduled BTKi pauses for other reasons, such as elective surgery, the declining humoral response on recommencement of BTKi in most patients suggests this may not be a worthwhile strategy to pursue a sustained immune response. Additionally, control of the patient's underlying haematological malignancy needs to be ensured as some studies have shown an increased risk of progressive disease associated with pauses in therapy [14].

## 5 CONCLUSION

A larger body of prospective data is warranted before any firm conclusions can be made and this study may lay a foundation for future research in maximising response to vaccination in this vulnerable patient cohort.

## CONFLICT OF INTEREST STATEMENT

The authors declare no conflict of interest.

## ETHICS STATEMENT

The study was approved by the Sydney Local Health District Human Research Ethics Committee and all subjects provided written informed consent prior to participation.

## Data Availability

The data that support the findings of this study are available on request from the corresponding author. The data are not publicly available due to privacy or ethical restrictions.

## References

[1] Visco C , Marcheselli L , Mina R , Sassone M , Guidetti A , Penna D , et al. A prognostic model for patients with lymphoma and COVID‐19: a multicentre cohort study. Blood. 2022;6(1):327–38. 10.1182/bloodadvances.2021005691 PMC8516438 34644385

[2] Owen R , Treon S , Al‐Katib A , Fonseca R , Greipp PR , McMaster ML , et al. Cliniclopathological definition of Waldenstrom's macroglobulinemia: consensus panel recommendations from the second international workshop on Waldenstrom's macroglobulinemia. Semin. Oncol. 2003 Apr;30:110–5. 12720118 10.1053/sonc.2003.50082

[3] Treon S . How I treat Waldenstrom macroglobulinemia. Blood. 2015 Aug 6;126(6):721–32. 26002963 10.1182/blood-2015-01-553974

[4] Tam CS , Ou YC , Trotman J , Opat S . Clinical pharmacology and PK/PD translation of the second generation Bruton's tyrosine kinase inhibitor, zanubrutinib. Expert review of Clinical Pharmacology. 2021 Nov;14(11):1329–44. 34491123 10.1080/17512433.2021.1978288

[5] Douglas A , Trubiano J , Barr I , Leung V , Slavin MA , Tam CS . Ibrutinib may impair serological responses to influenza vaccination. Haematologica. 2017 Oct;102(10):e397–9. 28659336 10.3324/haematol.2017.164285 PMC5622870

[6] Pleyer C , Ali MA , Cohen JI , Tian X , Soto S , Ahn IE , et al. Effect of Bruton tyrosine kinase inhibitor on efficacy of adjuvanted recombinant hepatitis B and zoster vaccines. Blood. 2021 Jan 14;13(2):185–9. 10.1182/blood.2020008758 PMC7820878 33259596

[7] Bagacean C , Letestu R , Al‐Nawakil C , Brichler S , Levy V , Sritharan N , et al. Humoral response to mRNA anti‐COVID‐19 vaccines BNT162b2 and mRNA‐1273 in patients with chronic lymphocytic leukemia. Blood Adv. 2022 Jan 11;6(1):207–11. 34844264 10.1182/bloodadvances.2021006215 PMC8632355

[8] Herishanu Y , Avivi I , Aharon A , Shefer G , Levi S , Bronstein Y , et al. Efficacy of the BNT162b2 mRNA COVID‐19 vaccine in patients with chronic lymphocytic leukemia. Blood. 2021 Jun 10;137(23):3165–73. 33861303 10.1182/blood.2021011568 PMC8061088

[9] Beaton B , Sasson S , Rankin K , Raedemaeker J , Wong A , Hastak P , et al. Patients with treated indolent lymphomas immunized with BNT162b2 have reduced anti‐spike neutralizing IgG to SARS‐CoV‐2 variants, but preserved antigen‐specific T cell responses. Am J Hematol. 2022 May 24;98(1):131–9. 35607995 10.1002/ajh.26619 PMC9349368

[10] Tomowiak C , Leblond V , Laribi K , Baron M , Puppinck C , Gerard P , et al. Response to vaccination against SARS‐CoV‐2 in 168 patients with Waldenstrom macroglobulinaemia: a French Innovative Leukaemia Organization study. Br J Haematol. 2022 May;197:424–7. 35029297 10.1111/bjh.18055

[11] Gavriatopoulou M , Terpos E , Ntanasis‐Stathopoulos I , Briasoulis A , Gumeni S , Malandrakis P , et al. Poor neutralizing antibody responses in 106 patients with WM after vaccination against SARS‐CoV‐2: a prospective study. Blood Adv. 2021 Nov 1;5(21):4398–405. 34529762 10.1182/bloodadvances.2021005444 PMC8450138

[12] Mateus J , Grifoni A , Tarke A , Sidney J , Ramirez SI , Dan JM , et al. Selective and cross‐reactive SARS‐CoV‐2 T cell epitopes in unexposed humans. Science. 2020 Oct 2;370(6512):89–94. 32753554 10.1126/science.abd3871 PMC7574914

[13] Tea F , Stella AO , Aggarwal A , Darley DR , Pilli D , Vitale D , et al. SARS‐CoV‐2 neutralizing antibodies: longevity, breadth, and evasion by emerging viral variants. PLoS Med. 2021 Jul 6;18(7):e1003656. 34228725 10.1371/journal.pmed.1003656 PMC8291755

[14] Castillo J , Gustine J , Meid K , Dubeau T , Xu L , Yang G , et al. Impact of ibrutinib dose intensity on patient outcomes in previously treated Waldenström macroglobulinemia. Haematologica. 2018 Oct;103(10):e466–e468. 29773590 10.3324/haematol.2018.191999 PMC6165799

